# Isokinetic Muscle Strength and Postural Sway of Recreationally Active Older Adults vs. Master Road Runners

**DOI:** 10.3389/fphys.2021.623150

**Published:** 2021-03-18

**Authors:** Henrique V. Taveira, Claudio A. B. de Lira, Marilia S. Andrade, Ricardo B. Viana, Hirofumi Tanaka, Lee Hill, Pantelis T. Nikolaidis, Beat Knechtle, Thomas Rosemann, Rodrigo L. Vancini

**Affiliations:** ^1^Centro de Educação Física e Desportos, Universidade Federal do Espírito Santo, Vitória, Brazil; ^2^Setor de Fisiologia Humana e do Exercício, Laboratório de Avaliação do Movimento Humano, Faculdade de Educação Física e Dança, Universidade Federal de Goiás, Goiânia, Brazil; ^3^Departamento de Fisiologia, Universidade Federal de São Paulo, São Paulo, Brazil; ^4^Department of Kinesiology and Health Education, The University of Texas at Austin, Austin, TX, United States; ^5^Division of Gastroenterology and Nutrition, Department of Pediatrics, McMaster University, Hamilton, ON, Canada; ^6^School of Health and Caring Sciences, University of West Attica, Athens, Greece; ^7^Medbase St. Gallen Am Vadianplatz, St. Gallen, Switzerland; ^8^Institute of Primary Care, University of Zurich, Zurich, Switzerland

**Keywords:** physical exercise, aging, isokinetic dynamometer, master athletes, road runners, postural balance, muscle strength

## Abstract

Trunk muscle strength and control is an important prerequisite for everyday activities among elderly people decreasing the predisposition to falls. High levels of physical exercise performed by older athletes could offer benefits to core/trunk muscle strength and postural control compared with recreational physical activities and among elderly people with lower levels of physical activity. The present study aimed to compare trunk muscle strength and postural control of older running athletes vs. older physically active adults. Participants were master road runners (RUN, *n* = 15, six women, 64.3 ± 3.6 years) and physically active elderly (control group, CON, *n* = 15, six women, 65.4 ± 5.0 years) people that were submitted to the evaluations: esthesiometer, posturography (force plate), and isokinetic test (Biodex dynamometer) of trunk muscle extension and flexion. RUN presented higher values for relative peak torque of trunk extensor muscles at 60°/s (*p* = 0.046) and 180°/s (*p* = 0.007) and relative average power during trunk extension at 60°/s (*p* = 0.008) and 180°/s (*p* = 0.004) compared to CON. CON had a higher medial-lateral oscillation speed of the center of pressure in the stable condition with eyes closed (*p* = 0.004) compared to RUN. RUN presented higher isokinetic torque of extensor trunk muscles and better postural control than CON. This supposedly could help with postural control and balance and contribute to the prevention of falls among the elderly. The practice of running systematically by master athletes may partially explained our findings.

## Introduction

Maintaining a high level of physical, psychological, cognitive, and social functions in the aging process, and later life is a critical component of successful aging (Geard et al., 2017). Master’s athletes are often considered examples of successful aging (Tanaka and Seals, 2008; Geard et al., 2017). This special population demonstrates impressive sports and physiological, mental vigor, muscle strength, and body balance performances (Tanaka and Seals, 2008). In recent years, the athletic performance of master road runners has been progressing rapidly, such that their performance is approaching closer to those of the youngest athletes (Akkari et al., 2015). However, scientific evidence shows that a large amount of strenuous physical activity is not necessarily required for older adults to maintain good health and quality of life (McPhee et al., 2016). However, for those people who want to compete in the more advanced stages of the aging process, age is not a limiting factor in itself (Tanaka and Seals, 2008; Akkari et al., 2015).

The aging process is commonly characterized by deteriorations in musculoskeletal functions, which result in functional declines in daily living activities, compounded with a subjective feeling of weakness and loss of coordination and balance, leading to frequent falls (Wroblewski et al., 2011). At 80 years of age, trunk muscle torque is reduced by up to 60% compared to young adults (Sasaki et al., 2018). Age-related deterioration and loss of muscle mass can be seen as young as 50 years of age, which may predispose a person to an increased risk of injury, e.g., rotator cuff disease (Han Oh et al., 2010). Therefore, one of the growing concerns in elderly adults that is under-studied and under-researched is muscle performance of core muscles, particularly trunk flexor, and extensor muscles (Wroblewski et al., 2011; Steele et al., 2016; Sasaki et al., 2018). Muscle performance (strength and power) of trunk extensor muscles is related to lumbar kinematics during walking (Steele et al., 2016) since the actions of trunk muscles contribute to the stability of the spine.

Physical exercise appears to modulate the morbidities of aging, preserving muscle mass and strength, and thus decreasing and/or even eliminating limits in independence (power of action) and autonomy (power of decision), losses of balance, and falls, that affect a significant proportion of the elderly population (Wroblewski et al., 2011). However, older adults who practice different types of physical exercise appear to preserve better balance and posture control and maintain levels of strength and muscle mass (Youssef and Abd elhameed Shanb, 2016). For example, older adults who play sports that promote disturbances of the postural control and sway patterns (for example, ice skating) exhibit postural control patterns more similar to young adults (Perrin et al., 1999; Tsang and Hui-Chan, 2005). That is, in a general way, regular physical exercise practice could minimize the detrimental and negative age-related changes in postural control and muscle strength (Lamoth and van Heuvelen, 2012). Additionally, older people that practice physical modalities such as *Tai Chi* present a better level of lower limb strength, reduced body oscillation, and greater confidence in balance (Tsang and Hui-Chan, 2005).

It is currently unknown whether high levels of physical exercise similar to those performed by older athletes provide additional benefits for postural control and core (trunk) muscle strength above and beyond recreational physical activities. No study is available to answer this question. Accordingly, the present study aimed to compare trunk muscle function, muscle performance, and postural control between older competitive master’s running athletes and age-matched physically active and non-competitive older adults. Considering the dose-responses relationship between physical exercise levels and musculoskeletal benefits, we hypothesized that isokinetic core (trunk) muscle strength and postural control of master older running athletes would be better than their physically active older peers.

## Materials and Methods

### Ethics Statement

All experimental procedures were approved (protocol number – 2.410.060) by the University Research Ethics Committee (Federal University of Espírito Santo – UFES) and followed the principles outlined in the Declaration of Helsinki.

### Participants

Fifteen older master’s running athletes (six women and nine men – RUN) and 15 recreationally active older adults (six women and nine men– control group, CON) participated in the study. The two groups were matched for sex, age, height, and body mass (Table 1) and contacted through coaches and physical trainers of master athletes and for convenience. Endurance running was the sole sporting modality for 20% of RUN (*n* = 3). For the remaining 80% (*n* = 12), at least one type of exercise and sport (resistance training, etc.) was performed in addition to endurance running. That is, 100% of RUN ran systematically. For the purposes of the present study, master’s athletes were defined by those people who participated in competitions and athletic events (Perrin et al., 1999; Vora et al., 2018). The CON group practiced regular physical activity (walking, dancing, resistance training, gymnastics, etc.), but did not participate in any sport at a competitive level and were recruited from a from gyms, dance groups, personal contacts, and through indication. The duration of physical activity practice, in a systematic way, for the CON and RUN groups ranged from 3 months or more to more than 5 years. The vast majority of people who participated in the study received specialized professional guidance for the practice of physical exercise and had no motor and/or physical mobility difficulties. As for health care, all participants were attended in a private and/or public health service for health care. The vast majority of participants, from both groups, reported that they did not use cigarettes and consumed little alcohol.

**TABLE 1:** General characteristics of older recreationally-active adults (CON) and older master road runners (RUN) groups.

	CON (*n* = 15)	RUN (*n* = 15)	*p*-value
Sex (males/females)	9/6	9/6	–
Age (years)	65.4 (5.0)	64.3 (3.6)	0.509
Body mass (kg)	66.9 (12.3)	63.3 (7.2)	0.324
Height (cm)	1.61 (0.10)	1.62 (0.08)	0.829
Body mass index (kg/m^2^)	25.7 (3.3)	24.1 (2.4)	0.158
Weekly frequency of exercise (days/weeks)	3 (2)^*a*^	5 (3)^*a*^	0.072
Physical activity (hours/weeks)	5 (3)^*a*^	6 (4)^*a*^	0.170
MMSE	26 (3)^*a*^	26 (3)^*a*^	0.733

*SD, standard deviation; MMSE, mini mental state examination. ^*a*^Data are presented as median (interquartile range).*

The following inclusion criteria were adopted: (i) at least 60 years of age; (ii) no diseases that affect balance control; (iii) no history of spinal surgery; (iv) no acute pain that would affect performance on the tests; (v) preserved cognitive functions evaluated by the mini-mental state examination (MMSE) (Brucki et al., 2003); (vi) the presence of plantar sensitivity in both feet (cut point, 10 g filament) (Mantovani et al., 2014); (vii) body mass index <30 kg/m^2^ because severe obesity impairs balance control in older adults (Frames et al., 2018), and (viii) in the case of RUN being practitioners and actively participating in road running competitions.

This cross-sectional study was conducted in a single visit for each participant and lasted approximately 3 h. All the experimental procedures were explained to the participants, and they provided written informed consent prior to participation.

### Experimental Procedures

A questionnaire composed of queries on sociodemographic data (14 questions), health conditions (18 questions), medications (six questions), and history of orthopedic injuries (five questions) was administered. The validated MMSE verified the cognitive functions of the participants for the Brazilian population. This test was only used for screening and characterization of the studied sample. The MMSE assesses cognitive impairment and can be used to detect cognitive impairment and monitor aging-associated diseases (Folstein et al., 1975). It includes five questions on temporal and spatial orientation; one question of immediate memory, attention and calculation, and evocation; and a task of appointment, repetition, fulfilling a command, reading, writing, and drawing copy. The score was the sum of the participant’s correct answers. Habitual physical activity was evaluated using the Baecke Physical Activity Questionnaire (Baecke et al., 1982) modified for older adults (Voorrips et al., 1991), and validated for Brazilian Portuguese (Ueno, 2013). In order to account for any potential issues relating to comprehension, an interview was used to fill out the questions. The total score for habitual physical activity was obtained by summing the physical exercise in leisure and leisure and locomotion activity scores. The higher the score, the greater the level of habitual physical activity.

Participants’ height and body mass were measured to the nearest 0.1 cm and 0.1 kg, respectively. Body mass index was calculated by dividing body mass by height squared (kg/m^2^). Older adults commonly demonstrate less plantar sensitivity compared with young adults. Interferences in posturography results can be minimized by ensuring that participants have good plantar sensitivity (Ueda and Carpes, 2013). To confirm this, cutaneous sensibility in the plantar region of the feet was evaluated using nylon monofilaments Semmes-Weinstein (SorriBauru^®^, Bauru, Brazil) of different diameters and equal length. The assessment included a bilateral standardized pressure on the skin in the regions of the sensory dermatomes and anterior tibial nerves. Insensitivity to the 10 g monofilament was adopted as the cut-off point for an exclusion criterion (Mantovani et al., 2014).

Postural control was assessed using a bipodal force plate (EMG system do Brazil Ltda., BIOMEC400-810) adjusted to 100 Hz in the signal acquisition frequency, with a signal filter set to 10 Hz (Donath et al., 2016). Participants were instructed to stand barefoot with their arms at their side and to keep their posture as still as possible. A point on the wall 150 cm away from the participant was used as a reference in the open-eyed condition (Cattagni et al., 2014). A viscoelastic foam (RM Products; 50 cm long × 40 cm wide × 6.5 cm high) positioned on the force plate was used to generate unstable surface conditions (Yahia et al., 2011). Thus, four test conditions were performed, which varied between a stable and unstable surface with open and closed eyes, all performed three times for 30 s each with a 1-min interval between the tests. The condition order was randomized. The mean value of the three trials for each of the four test conditions was used in the data analyses.

The stabilogram displays the coordinates of the center of pressure as a function of time. Center of pressure variations were analyzed in amplitude and velocity of center of pressure displacements in the mediolateral (mediolateral/*x*-coordinate) and anteroposterior (anteroposterior/*y*-coordinate) directions, as well as the elliptic area of center of pressure displacement (Prasertsakul et al., 2018). The amount of center of pressure variation in a quiet posture is related to the balance for maintenance of the projected center of mass within the support base. Greater variations indicate lower postural stability (Prasertsakul et al., 2018).

Core muscle performance (strength and power) of the trunk extensor and flexor muscles was performed using an isokinetic dynamometer (Biodex System 4 Pro^TM^, Biodex Medical Systems, Shirley, NY, United States). Participants adopted the seated-compressed position (isolated lumbar position), with thighs and hip fixed by strips and the trunk properly attached to the equipment, allowing only for trunk extension/flexion movements. This setup controls the involvement of the hip muscles (particularly the iliopsoas) during flexion and the hip extensor muscles during trunk extension (Morini et al., 2008). The anterior superior iliac spine was aligned with the axis of the equipment through adjustments in the modulus. Data generated with the anterior-superior iliac spine alignment are more consistent in the expected velocity-to-torque ratios than other parameters and are associated with the lowest overall variability of the data, even if the tests do not show definite statistical evidence of the superiority of one alignment compared to the others (Grabiner et al., 1990). Familiarization occurred from two submaximal attempts at a 60°/s angular speed.

Range of motion was determined subjectively according to participants’ level of discomfort for trunk flexion/extension. The test protocol consisted of a series of five repetitions at 60°/s followed by 15 repetitions at 180°/s. Both series were performed in the CONCENTRIC/CONCENTRIC mode for trunk flexion/extension, respectively, and with a 60 s interval between them. The variables evaluated were peak torque (in Nm), average power (in watts), and the flexor/extensor torque ratio (%) at both angular speeds. This relationship was assessed because musculoskeletal imbalance was associated with a higher incidence of low back pain (Ripamonti et al., 2011). Peak torque and mean power values were normalized by total body mass. All participants received instructions about the isokinetic assessment and standardized verbal encouragement during the test in order to encourage maximum performance. The isokinetic trunk test was performed last to avoid any residual fatigue effects on postural control tests.

### Statistical Analyses

The Shapiro–Wilk test was used to verify the normality of the data distribution. Age, body mass, height, body mass index, peak torque isokinetic data for trunk extensor and flexor muscles at 60 and 180°/s, average trunk extensor power at 60°/s, average trunk flexors power at 180°/s, the agonist/antagonist ratio at 60 and 180°/s, postural velocity control mediolateral data with open eyes, anteroposterior velocity and mediolateral with closed eyes (on a rigid surface), anteroposterior velocity with open eyes, anteroposterior amplitude and mediolateral amplitude, area, and anteroposterior velocity with closed eyes (on an unstable surface) were normally distributed and tested with the *t*-test for unpaired samples. The other data were tested with the Mann–Whitney U test because they were not normally distributed. The level of significance adopted in all analyses was 5%, with a 95% confidence interval. All data were analyzed through the Statistical Package for the Social Sciences (SPSS, version 25.0, IBM Corp., Armonk, NY, United States). Cohen’s *d* effect size measures for differences were calculated by dividing the mean change between groups by the pooled group standard deviation. For the non-normal data, Cohen’s *d* effect size measures for differences were estimated from Mann–Whitney analysis using the following formula (Gignac, 2019): $d=Z\,x\,\sqrt{1/N_{1}}+1/N_{2}$. According to Cohen, the values of “*d*” were classified according to the following criteria: *d* < 0.2 was considered “trivial,” 0.2 ≤ *d* < 0.5 was considered “small,” 0.5 ≤ *d* < 0.8 represented “medium,” and *d* ≥ 0.8 constituted “large” (Cohen, 1988). Data are presented as mean ± standard deviation or median and interquartile range, and changes (Δ – delta) and 95% confidence interval unless otherwise stated.

## Results

### Habitual Physical Activity Assessment

The habitual physical activity level was significantly higher in RUN compared with CON [median: 6.6 (95% CI: 1.5; 11.6); *p* = 0.010; *d* = 0.95 (large effect)].

### Postural Control Assessment

In the evaluation at force plate, there were no significant group differences for both stable and unstable conditions with open or closed eyes for any of the variables, except for mediolateral velocity in the stable condition with closed eyes, in which CON showed significant higher (*p* = 0.004) oscillation in center of pressure than RUN (Table 2).

**TABLE 2:** Profile of postural control variables of older recreationally active adults (CON, *n* = 15) and older master road runners (RUN, *n* = 15) groups.

	CON	RUN	Δ (95% CI)	%	*p*	Cohen’s *d*
	Mean (SD)	Mean (SD)				
**Stable surface**						
*Eyes open*						
Anteroposterior amplitude (cm)	1.1 (0.4)^*a*^	1.0 (0.4)^*a*^	Median: 0.1 (−0.2; 0.4)	10.0	0.389	0.33(small)
Mediolateral amplitude (cm)	1.8 (0.6)^*a*^	2.0 (0.6)^*a*^	Median: −0.1 (−0.5; 0.2)	−10.0	0.325	−0.37(small)
Elliptic/sway area (cm^2^)	1.1 (0.9)^*a*^	1.6 (1.3)^*a*^	Median: −0.3 (−1.0; 0.3)	−31.3	0.187	−0.49(small)
Anteroposterior velocity (cm/s)	0.8 (0.3)^*a*^	0.9 (0.3)^*a*^	Median: −0.1 (−0.4; 0.1)	−11.1	0.305	−0.38(small)
Mediolateral velocity (cm/s)	1.4 (0.3)	1.2 (0.2)	0.15 (−0.05; 0.3)	16.7	0.132	0.57(medium)
*Eyes closed*						
Anteroposterior amplitude (cm)	1.2 (0.2)^*a*^	1.2 (0.5)^*a*^	Median: 0.0 (−0.3; 0.2)	0.0	0.653	0.17(trivial)
Mediolateral amplitude (cm)	2.0 (0.4)^*a*^	2.5 (1.0)^*a*^	Median: −0.3 (−0.9; 0.1)	−20.0	0.187	−0.48(small)
Elliptic/sway area (cm^2^)	1.2 (0.9)^*a*^	1.8 (1.6)^*a*^	Median: −0.5 (−1.4; 5.2)	−33.3	0.050	−0.72(medium)
Anteroposterior velocity (cm/s)	0.9 (0.2)	0.9 (0.2)	−0.1 (−0.2; 0.1)	0.0	0.357	−0.34(small)
Mediolateral velocity (cm/s)	1.6 (0.3)	1.3 (0.2)	0.3 (0.1; 0.5)	23.1	0.004	1.16(large)
**Unstable surface**						
*Eyes open*						
Anteroposterior amplitude (cm)	2.7 (0.4)^*a*^	2.7 (1.1)^*a*^	Median: −0.1 (−0.8; 0.3)	0.0	0.713	−0.14(trivial)
Mediolateral amplitude (cm)	4.1 (1.0)^*a*^	3.6 (1.8)^*a*^	Median: 0.3 (−0.8; 1.1)	13.9	0.567	0.22(small)
Elliptic/sway area (cm^2^)	7.4 (2.4)^*a*^	6.8 (5.0)^*a*^	Median: 0.3 (−3.5; 1.9)	8.8	0.775	0.11(trivial)
Anteroposterior velocity (cm/s)	1.5 (0.4)	1.4 (0.2)	0.1 (−0.2; 0.3)	7.1	0.569	0.21(small)
Mediolateral velocity (cm/s)	1.7 (0.5)^*a*^	2.0 (0.7)^*a*^	Median: −0.1 (−0.5; 0.2)	−15.0	0.595	−0.20(small)
*Eyes closed*						
Anteroposterior amplitude (cm)	4.2 (0.9)	4.1 (0.8)	0.1 (−0.5; 0.8)	2.4	0.693	0.15(trivial)
Mediolateral amplitude (cm)	6.5 (2.0)	5.7 (1.0)	0.8 (−0.4; 2.0)	14.0	0.193	0.49(small)
Elliptic/sway area (cm^2^)	17.4 (8.2)	14.9 (4.0)	2.5 (−2.3; 7.3)	16.8	0.304	0.39(small)
Anteroposterior velocity (cm/s)	2.2 (0.6)	2.1 (0.5)	0.05 (−0.4; 0.5)	4.8	0.823	0.08(trivial)
Mediolateral velocity (cm/s)	2.9 (0.6)^*a*^	3.3 (1.1)^*a*^	Median: −0.3 (−0.9; 0.1)	−12.1	0.174	−0.51(medium)

*SD, standard deviation; CI, confidence interval. ^*a*^Data are presented as median (interquartile range).*

### Isokinetic Core Muscle Performance (Peak Torque and Power)

There were no significant group (RUN vs. CON) differences in relative peak torque of trunk flexion at 60 and 180°/s, relative power of trunk flexion at 60 and 180°/s, and agonist/antagonist peak torque ratio at 60 and 180°/s (Table 3). However, RUN presented a greater relative peak torque of trunk extension at 60°/s (*p* = 0.046), and a higher relative peak torque of trunk extension at 180°/s (*p* = 0.007), and a higher relative power of trunk extension at 60 (*p* = 0.008) and 180°/s (*p* = 0.004) compared with CON (Table 3).

**TABLE 3:** Profile of isokinetic trunk variables of older recreationally active adults (CON, *n* = 15) and older master road runners (RUN, *n* = 15) groups.

	CON	RUN	Δ (95% CI)	%	*p*	Cohen’s *d*
	Mean (SD)	Mean (SD)				
Peak torque of extensor at 60°/s (Nm/kg)	2.6 (0.7)	3.1 (0.7)	−0.6 (−1.1; 0.3)	–16.1	0.046	−0.76(medium)
Peak flexor torque at 60°/s (Nm/kg)	1.4 (0.5)	1.7 (0.5)	−0.2 (−1.1; 0.1)	–17.6	0.127	−0.58(medium)
Peak extensor torque at 180°/s (Nm/kg)	2.7 (0.7)	3.6 (0.9)	−0.8 (−1.4; −0.3)	–25.7	0.007	−1.07(large)
Peak flexor torque at 180°/s (Nm/kg)	1.9 (0.6)	2.2 (0.6)	−0.2 (−0.7; 0.2)	–13.6	0.338	−0.36(small)
Average extensor power at 60°/s (W/kg)	1.1 (0.6)	1.7 (0.5)	−0.6 (−1.0; −0.2)	–35.3	0.008	−1.04(large)
Average flexor power at 60°/s (W/kg)	0.7 (0.5)^*a*^	1.0 (0.5)^*a*^	Median: −0.2 (−0.5; 1.4)	30.0	0.067	−0.67(medium)
Average extensor power at 180°/s (W/kg)	0.9 (0.8)^*a*^	1.9 (1.8)^*a*^	Median: −0.7 (−1.9; −0.2)	52.6	0.004	−1.02(large)
Average flexors power at 180°/s (W/kg)	0.9 (0.5)	1.2 (0.5)	−0.3 (−0.73; 0.03)	–25.0	0.072	−0.68(medium)
Flexors/extensors peak torque ratio at 60°/s (%)	56 (18)	55 (14)	1.7 (10.3; 13.8)	1.8	0.771	0.11(trivial)
Flexors/extensors peak torque ratio at 180°/s (%)	72 (20)	62 (11)	10.4 (1.5; −22.2)	16.1	0.083	0.66(medium)

*SD, standard deviation; CI, confidence interval. ^*a*^Data are presented as median (interquartile range).*

## Discussion

The present study aimed to compare isokinetic trunk strength and balance of master’s road runners vs. older recreationally and non-competitive physically active adults. Master’s road runners presented significantly greater values for relative peak torque and relative power, trunk extensor muscles, at 60 and 180°/s, and lower medial-lateral oscillation speed of the center of pressure in the stable condition with eyes closed when compared to older recreationally and non-competitive physical active adults. These results suggest that lifelong and higher levels of physical exercise could help with postural control and could contribute to preventing falls and maintaining functional capacity at high levels among the elderly. For example, Choi et al. (2016) showed that muscle strength and standing balance are reflected in physical balance ability and the possible prevention of falls in the elderly people (fallers, non-fallers, healthy balance group, and impaired balance group) who had high levels of physical activity and consequently improve the functional capacity. Thus, the results of our study reinforces that elderly athletes who participate in sports and in competitions regularly can be considered a successful model of aging which is somewhat new since our study focused on the musculature of the trunk and not the lower limbs as traditionally done.

Recently, an elegant review article of Ben Moussa Zouita et al. (2020) established normative data and profile, for the elderly people, regarding to the isokinetic trunk muscle strength. However, normative data for elderly athletes (and in the case of our study, practitioners, and competitors in road racing events) are lacking. This was one of the motivators for carrying out this study, in addition to revealing what may differentiate a less active elderly person from a more physically active one. Both groups (RUN and CON) demonstrated similar postural controls, except for a significant difference in the mediolateral velocity in the stable condition with closed eyes whose oscillation was greater for CON. In addition, isokinetic trunk muscle performance during extension (but not for flexion) at 60 and 180°/s was greater in RUN compared with CON.

Similar findings have been reported in younger high-level athletes (recruited from boxing, wrestling, and weightlifters; modalities which require much more strength from the trunk muscles) who exhibited greater muscle performance for trunk extensor muscles, but not for flexor muscles, when compared with control group composed by recreationally physical active people (Ben Moussa Zouita et al., 2018).

In our study, the group of elderly athletes was 100% composed of people who practiced and competed in road running events. This may have had a direct influence on our findings. In this context, biomechanical studies on running have shown that trunk flexion in the sagittal plane is a common pattern among runners (Teng and Powers, 2014, 2015), resulting in a significant influence on the absorption and generation of energy from the hip and knee (Teng and Powers, 2015). Increasing the inclination of the trunk forward during running is a strategy to reduce the load imposed on the knee joint (Teng and Powers, 2015). Together, these biomechanical patterns assumed during running generate greater activation of the trunk extensors and, consequently, assist in the strengthening of these muscles.

Physiologically, there is evidence of the preservation of motor units (and consequently muscle strength) within muscles with greater specificity during the running action, for example, anterior tibialis of elderly runners (with an average age close to our study – 64 years) (Power et al., 2010). However, it has been shown that the same does not happen in muscles without this specific action during running, for example, biceps brachii (Power et al., 2012). Additionally, it is acceptable to assume that the spine plays a decisive role in absorbing shock derived from the ground force reaction (Simoni et al., 2020). The requirements for trunk muscle actions are specific to each type of physical effort and can induce different muscle adaptations (Ben Moussa Zouita et al., 2020) and it seems to have some relation to the age of the sample. However, further studies are needed to assess how much the biomechanical running pattern affects the activation and strengthening of trunk muscles among elderly running athletes.

A similar pattern was also observed in other studies, with higher values of peak torque and isokinetic power in extension compared to trunk flexion at speeds of 60 and 180°/s, in young people (Bernard et al., 2014) and older/elderly people and healthy younger adults and those with chronic low-back pain (Shirado et al., 1992; Yahia et al., 2011). In general, in isokinetic evaluation, higher values are observed for the performance of trunk extensor muscles than for flexors in the sagittal plane (Ben Moussa Zouita et al., 2020) with rare exceptions (Mueller et al., 2012); for example, in adults with low back pain (Yahia et al., 2011). The average of the concentric torques at 60°/s of extension and flexion of the trunk of healthy adults of both sexes is 208 Nm (range: 121–360 Nm) and 176 Nm (range, 111–296 Nm), respectively, with a flexion ratio of the trunk for extension of 0.84 (variation, 0.54–1.16) (Mueller et al., 2012).

It is noteworthy that the ratio of isokinetic peak torque to trunk flexion/extension provides valuable information on muscle balance and performance (Yahia et al., 2011) because changes in this proportion raise the value of the trunk flexors/extensors ratio and are associated with low back pain (Ripamonti et al., 2009; Yahia et al., 2011; Mueller et al., 2012). In healthy adults, the trunk flexor/extensor ratio is considered normal when less than 1, ranging from 0.80 to 0.85 (Gremion et al., 1996; Yahia et al., 2011; Mueller et al., 2012). In athletes, these values tend to be lower (Ben Moussa Zouita et al., 2020). Healthy adult athletes had the highest values of trunk strength, but the lowest flexor/extensor ratio, with the reference value at 60°/s being 0.62 for both sexes (Mueller et al., 2012). The values found in this study in both groups are below the reference values for healthy adults and close to the reference value for adult athletes (0.62) and also for physically active young adults (0.66) and young adult athletes (0.59) (Ben Moussa Zouita et al., 2018).

In our study, the ratio between trunk flexors/extensors was 0.56–0.72 for CON and 0.55–0.62 for RUN at 60 and 180°/s, respectively. An intervening factor for the values of the flexion and extension ratio found in this study may be the average age of the groups that is, RUN 64.3 years and CON 65.4 years. It has already been demonstrated that, when compared with young adults, the peak torque for trunk flexion decreases after 60 years of age and the extension torque later on, after 70 years of age in both sexes (Sasaki et al., 2018).

Regarding the variables of postural control, obtained through evaluation in force plate, significant differences were found in relation to the variable mid-lateral speed in the stable condition with eyes closed, as RUN showed less oscillation (23.1%) than the center of pressure. It is important to note that postural control with eyes closed depends to a greater degree on the accuracy of body kinesthetic information, proprioception mechanisms, and the inner ear, as well as triggering reflex modulations in the activation of postural muscles in an efficient manner given the absence of visual feedback (Leightley et al., 2017). However, positive effects evoked by regular physical activity on postural control are related to the type of physical activity, test protocol, and the assessed posturography variables (Hugel et al., 1999; Perrin et al., 1999; Asseman et al., 2004).

Our results indicate that physically active older people have this postural control system possibly more preserved from the effects of the aging process. Therefore, it is possible that there is an improved functioning, due to the systematic practice of running (100% of RUN of the present study) of the proprioception mechanisms involved in the static postural control of elderly athletes when compared to the elderly who are less physically active with regard to the condition without visual feedback as observed in previous research (Glofcheskie and Brown, 2017; Paillard, 2019). However, it is necessary to deepen the research on the possible interrelationships between postural skills and motor skills (sports), to unveil mechanisms related to the plasticity of postural function for motor and sports experience (Paillard, 2019), and to consider the process of aging. In our study, all the people in RUN were regular runners, which may have impacted our findings. It should be noted that Leightley et al. (2017) demonstrated that master running athletes have the same postural oscillation as young adults when in bipedal and unipodal conditions with eyes open. However, they show less oscillation when compared to the elderly to the less physically active elderly. It has also been observed that master athletes are more efficient in postural corrections in conditions of rapid disturbance and high speed of displacement of the center of gravity (Brauer et al., 2008).

In addition, Granacher et al. (2013a) pointed out that traditionally the assessment and training of balance, strength resistance, and muscle strength of the lower extremities are used to positively adjust muscle deficits associated with age and falls. However, there are few studies on the effects of strength training and strength resistance of postural and trunk muscles for the same purpose and which translate into the improvement of postural balance and muscle strength for the performance of functional tasks and activities of daily living and preventing falls among the elderly. Training aimed at improving the strength of the trunk muscles appears to improve static and dynamic balance and functional performance and prevent falls and could improve functional capacity.

The importance of studying the muscles of the upper extremity is confirmed by Granacher et al. (2013b) who demonstrated that age-related deficits in measures of trunk muscle strength, spinal mobility, dynamic balance and functional mobility can be mitigated by core instability strength training since these programs use exercises that are challenging for both trunk muscles and postural control and may thus have the potential to induce benefits in trunk muscle strength, spinal mobility, and balance performance and assist in preventing falls among the elderly. This core training regimen could be used as an adjunct or even alternative to traditional balance and/or resistance training to mitigate the effects of aging on generalized sarcopenia in both the lower and upper limbs.

Therefore, core and trunk musculature training should be used as a complement and alternative to traditional postural balance training programs for the elderly, who are mostly focused on the lower limbs (Muehlbauer et al., 2015). This denotes the importance of a study like ours that aims to broaden the vision for the importance of other muscles, as the muscular strength profile of the trunk and the effects of aging, in the body.

Thus, it is possible to conclude with our research that elderly athletes who practice running have greater strength and balance of the trunk muscles as well as less postural oscillation than elderly people who are less physically active. One can assume that with this scenario, there may be a protective effect in relation to falls, although it was not an objective to study falls among the elderly in our study. The fact of having better physical fitness related to health, that is, better balance and muscle strength, could exercise protection and prevent falls among the elderly. It must be remembered that falls among the elderly have harmful effects on their morbidity and mortality. Khow and Visvanathan (2017) pointed out that the aging process increases the risk of falls among elderly people. The negative impacts of falls among the elderly are injuries, hospitalizations, fear of falling, loss of independence, institutionalization, increased morbidity, and death.

Therefore, we can assume that the elderly athlete can be considered a successful model of aging and that maintaining and improving physical fitness through the practice of physical exercise during the aging process can resist its detrimental effects and possibly be seen as a “fountain of youth.”

## Study Limitations

This study is not without limitations. First, this was a cross-sectional study; therefore, longitudinal studies are needed to elucidate the long-term effect of physical training on postural variables. Second, weekly physical exercise frequency and exercise volume were not different between the groups based on the Baecke Questionnaire. In addition, exercise intensity was not assessed. However, it is reasonable to assume that RUN performed exercise sessions with greater intensity than those in the recreationally active group. Also, maximum aerobic capacity and power were not evaluated through maximum oxygen consumption. This could help in classifying the RUN and CON in terms of fitness level. Nevertheless, we believe that these limitations do not prevent conclusions from being drawn from the study.

## Perspectives

Health professionals could use these findings to counsel their older adult people regarding the benefits of regular physical exercise since we demonstrated that a large amount of physical exercise is not necessary to experience the benefits of physical activity on postural balance. For example, it has been demonstrated that intervention with physical exercises that involve dynamic postural stability control and require a high level of muscle strength production, in old adults (65–80 years), has the potential to enhance muscle strength and sensory information processing during sudden and static balance tasks and could reduce the risk of falls in older adults (Hamed et al., 2018). However, within the dose-response relationship between exercise and physical fitness, apparently, the group of older running athletes had a higher volume and intensity of physical training. This had a positive impact on muscle strength and balance. It is reasonable to assume that the practice of running competitively by the older master’s athlete group may have contributed to our findings. In addition, the division of groups using maximum aerobic power can be an interesting way for a future study to verify our findings. Also, future studies should focus on dynamic tasks of balance disturbance or on more complex tasks if they are comparing groups of master athletes and/or active healthy elderly.

Finally, it is also necessary to know well the physical activities of daily life (leisure, occupational, and sports), performed by people aged 60 or over, regarding the amount, intensity, frequency, and duration in order to have a clearer picture of the impact of the level of physical activity on the levels of functional capacity.

## Data Availability Statement

The raw data supporting the conclusions of this article will be made available by the authors, without undue reservation.

## Ethics Statement

The studies involving human participants were reviewed and approved by all experimental procedures were approved (protocol number – 2.410.060) by the University Research Ethics Committee (Federal University of Espírito Santo – UFES) and followed the principles outlined in the Declaration of Helsinki. The patients/participants provided their written informed consent to participate in this study.

## Author Contributions

HVT collected the data. HVT, RLV, CABL, MSA, and RBV conceived, designed, and performed the search strategy, performed the data collection and statistical analysis, and drafted, wrote, edited, and revised the manuscript. RLV, HT, CABL, MSA, LH, PTN, TR, and BK drafted, wrote, edited, and revised the manuscript. All authors contributed to the article and approved the submitted version.

## Conflict of Interest

The authors declare that the research was conducted in the absence of any commercial or financial relationships that could be construed as a potential conflict of interest.
